# The Influence of Working Memory Load on Expectancy-Based Strategic Processes in the Stroop-Priming Task

**DOI:** 10.3389/fpsyg.2017.00129

**Published:** 2017-02-01

**Authors:** Juan J. Ortells, Dolores Álvarez, Carmen Noguera, Encarna Carmona, Jan W. de Fockert

**Affiliations:** ^1^Department of Psychology, University of AlmeríaAlmería, Spain; ^2^Department of Psychology, Goldsmiths, University of LondonLondon, UK

**Keywords:** working memory load, cognitive control resources, Stroop interference, Stroop priming effects, expectancy-based strategic processes

## Abstract

The present study investigated whether a differential availability of cognitive control resources as a result of varying working memory (WM) load could affect the capacity for expectancy-based strategic actions. Participants performed a Stroop-priming task in which a prime word (GREEN or RED) was followed by a colored target (red vs. green) that participants had to identify. The prime was incongruent or congruent with the target color on 80 and 20% of the trials, respectively, and participants were informed about the differential proportion of congruent vs. incongruent trials. This task was interleaved with a WM task, such that the prime word was preceded by a sequence of either a same digit repeated five times (low load) or five different random digits (high load), which should be retained by participants. After two, three, or four Stroop trials, they had to decide whether or not a probe digit was a part of the memory set. The key finding was a significant interaction between prime-target congruency and WM load: Whereas a strategy-dependent (reversed Stroop) effect was found under low WM load, a standard Stroop interference effect was observed under high WM load. These findings demonstrate that the availability of WM is crucial for implementing expectancy-based strategic actions.

## Introduction

Working memory (WM) is the cognitive system that allows people to retain access to a limited amount of information, often in the service of complex cognition. There is growing evidence that WM plays a role in maintaining goal-directed behavior in the presence of potential distractors or contextually inadequate alternative responses. In order that our behavior can be successfully directed toward task-relevant information, both the target and competing distractors have to remain clearly separated in processing. WM has been proposed to be fundamental in this process (Lavie et al., 2004), and specifically in selective attention, which involves maintaining a goal-directed focus on one aspect of the environment (the relevant stimulus), while ignoring irrelevant aspects. Effective selection has been suggested to require both increased processing of the relevant information (facilitation), and active blocking or inhibition of irrelevant distractors, processes that are central to cognitive control and conflict resolution (Petersen and Posner, 2012).

Indirect evidence that WM is involved in achieving selective processing has initially come from studies on cognitive aging. There is ample evidence that WM performance deteriorates with age (e.g., see Gazzaley, 2012 for a review). At the same time, it has been shown that elderly participants are disproportionally impaired compared to younger participants at selective attention tasks that require active rejection of distracting information (e.g., De Fockert, 2005; De Fockert et al., 2009; see Zanto and Gazzaley, 2014, for a review).

More direct evidence for an association between WM and selective attention has come from two different lines of investigation. A first line of investigation uses a methodological strategy based in “extreme-groups,” in which WM capacities of a large sample of participants are assessed by means of several complex-span WM tasks (e.g., operation span; symmetry span). In a next phase, participants showing higher and lower scores (e.g., first vs. fourth quartiles) on WM tasks perform different selective attention tasks (e.g., Stroop; Eriksen-type flanker; Negative Priming). Individuals with high WM capacity are typically more effective at selectively attending to relevant, and overcoming the influence of irrelevant information, compared to individuals with low WM capacity. For instance, during the Stroop or Eriksen flanker tasks, low WM capacity individuals are more prone to interference from the irrelevant attribute of the stimulus than those with high WM capacity (e.g., Kane and Engle, 2003; Ahmed and De Fockert, 2012). In a similar vein, participants with greater WM capacity are also more efficient at actively ignoring irrelevant information in negative priming tasks (e.g., Conway et al., 1999; Ortells et al., 2016). Based on these findings, Engle and Kane (2004) and Kane et al. (2007) have proposed the attention control theory of WM, which states that individual differences in WM capacity mainly reflect variation in a domain-general attention control ability. This attention ability would be needed to actively maintain and recover access to task-relevant representations (e.g., sustain the task goal and constrain the focus of attention to relevant target items), and block access to task-irrelevant information.

A second line of evidence for a role of WM in selective attention uses a paradigm that combines a WM task with a selective attention task (e.g., Stroop) to measure distractor interference in a context of varying WM load (e.g., maintaining in memory one vs. six digits; see De Fockert, 2013, for a recent review). In a task requiring participants to classify a target name (popstar vs. politician) while ignoring either congruent or incongruent distractor faces (Young et al., 1986; De Fockert et al., 2001), any processing of the irrelevant faces would lead to poorer performance when the face category was incompatible with the current target name category, compared with trials on which the name and face categories were either compatible or unrelated (e.g., the name of a popstar with an anonymous face). When participants performed the selective attention task at the same time as a WM task of either low or high WM load, they showed greater Stroop-interference effects (in terms of both reaction times and accuracy rates) under high compared to low WM load (De Fockert et al., 2001). In addition, activation in brain areas dedicated to processing the irrelevant faces was also greater under high than under low WM load. Similar modulations of distractor processing as a function of WM load have been reported in a range of other selective attention tasks (e.g., Lavie and De Fockert, 2005; De Fockert et al., 2010). According to the load theory of attention by Lavie et al. (2004), WM load would deplete limited-capacity cognitive resources that are required to maintain goal distinctions between processed relevant and irrelevant information. Consequently, behavior would be more susceptible to be influenced by irrelevant information when WM load is high.

Whereas the work summarized above clearly demonstrates that a reduction in the availability of WM is associated with a greater difficulty for to actively reject and overcome the influence of distracting information that competes directly with target processing in selective attention tasks, much less is known about whether variations in the availability of WM could also influence on the efficacy of expectancy-based facilitatory strategies. Some recent semantic priming studies (Heyman et al., 2014; Hutchison et al., 2014) report evidence that under conditions that encourage the use of controlled strategies like expectancy generation (i.e., a high relatedness proportion), semantic priming effects are greatly reduced (or even eliminated) by imposing a high WM load, or for participants showing a low WM capacity.

Note, however, that these studies used a conventional facilitation paradigm, whereby controlled processes (e.g., expectancy generation) produce the same pattern (i.e., facilitation in performance) as do automatic processes (e.g., spreading activation). In other words, both the standard semantic priming effect and expectancy effects are expressed in terms of better performance for semantically related prime and probe information. Because both types of processes contribute to performance in a similar vein (i.e., facilitating), it is difficult to determine whether the reduced priming effects observed in individuals with low WM capacity, or under high WM load conditions, should be attributed to a reduction in automatic prime processing, or a less efficient (or delayed) use of expectancy-based strategies.

An alternative priming task which allows to obtain qualitatively different (i.e., opposite) behavioral effects promoted by controlled vs. automatic processes was used by Froufe et al. (2009) in a group of healthy young adults and two groups of elderly people, one with and one without Alzheimer’ dementia (AD). Participants performed a Stroop-priming task in which a prime word (GREEN or RED) was followed (after 1125 ms) by a colored (red vs. green) patch target that they had to identify. The prime was incongruent or congruent with the target color on 84 and 16% of the trials, respectively, and participants were informed about the differential proportion of congruent vs. incongruent trials. An opposite Stroop-priming pattern as a function of age was found. The young participants showed a reversed Stroop effect (i.e., faster responses on incongruent than on congruent trials), thus demonstrating that they were able to use the prime word in a strategic manner to anticipate the target color. In older people without AD the Stroop effect was also reversed, but not non-significantly so. In clear contrast, the older people with AD showed the opposite effect, namely a standard Stroop interference effect (i.e., slower responses on incongruent than on congruent trials), thus suggesting that AD is associated with additional loss (to that produced by advanced age) of the capacity for expectancy-based strategic actions.

Note, however, that the above evidence for a link between the availability of WM and strategic selective attention is indirect, as a reduction in WM capacity in the older (vs. younger) groups was assumed rather than measured. The main aim of the current study is therefore to obtain more direct evidence for an association between strategic controlled processes in selective attention and WM. A sample of young participants performed a Stroop-priming task similar to that used by Froufe et al. (2009; see also Merikle and Joordens, 1997; Daza et al., 2002). They had to identify the color (red vs. green) of a target stimulus (a series of ampersands in red vs. green), which was preceded by either a congruent or incongruent prime word (RED VS. GREEN), with incongruent prime-target trials being much more frequent (80%) than congruent prime-target pairs (20%). This Stroop-priming task was interleaved with a WM task of either low or high load. To the extent that expectancy-based strategic processes depend on the availability of cognitive control resources, we expected to obtain a reliable reversed Stroop-priming effect (i.e., faster responses on incongruent relative to congruent trials) similar to that observed by Froufe et al. (2009) in the younger group, but only under low WM load. In contrast, performing the Stroop-priming task under high WM load should give rise to a standard Stroop-interference effect (i.e., slower responses on incongruent relative to congruent trials), similar to that reported by Froufe et al. (2009) in the older with AD group.

## Materials and Methods

### Participants

Twenty-six healthy, right-handed, undergraduate students (16 women) from the University of Almería participated in a single experimental session, in exchange for course credit. Sample size was similar to previous studies on strategic priming (e.g., Merikle and Joordens, 1997; Froufe et al., 2009). All reported normal or corrected-to-normal vision, and were aged between 19 and 30 years (*M* = 22.35, *SD* = 2.8). This study was carried out in accordance with the recommendations of ‘Code of Good Practices in Research, Commission on Bioethics in Research from the University of Almería,’ with written informed consent from all subjects. All subjects gave written informed consent in accordance with the Declaration of Helsinki. The protocol was approved by the ‘Committee on Bioethics in Human Research’ from the University of Almería.

### Stimuli and Apparatus

**FIGURE 1:**
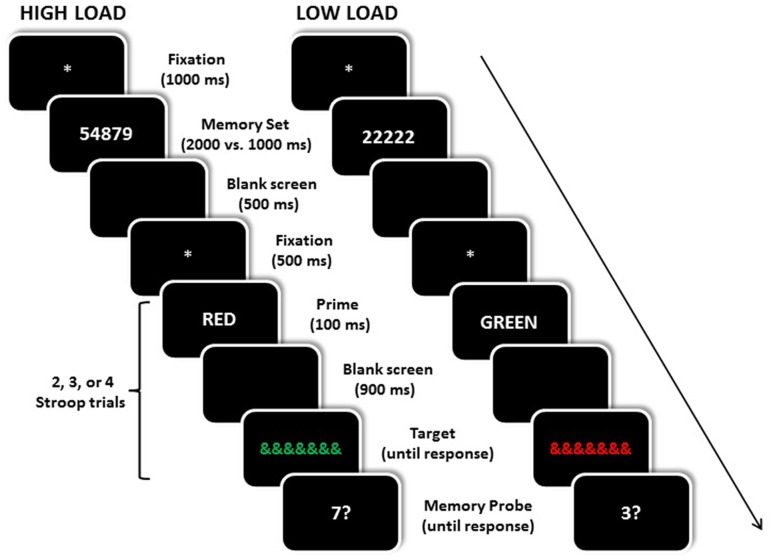
**Examples of the temporal sequence of events for an incongruent trial under high (left) and low (right) working memory load**. The prime words shown here have been translated from Spanish to English. Stimuli are not drawn to scale.

The experiment was run in a dimly lit testing cubicle on a PC running E-Prime software v2.0 (Psychology Software Tools, Pittsburgh, PA, USA). Stimuli were displayed on a 17inch CRT monitor at a viewing distance of approximately 60 cm. Responses were collected using a standard keyboard. The experiment trials consisted of an attention (Stroop-priming) and a WM (digit recall) component (see **Figure 1** for sample trial sequences). For the WM component, a five-digit set consisting of digits between 1 and 9 was displayed (in white) in Times New Roman font size 16 in the center of the screen. In the high WM load condition, the five digits were in a random non-sequential order (e.g., 81652). In the low WM load condition, the five digits consisted on a same digit repeated five times (e.g., 55555). The memory probe was a single white digit presented at fixation. Each digit subtended about 0.35° horizontally and 0.52° vertically. For the Stroop-priming component, two color words (RED or GREEN) displayed in white color were used as prime stimuli, with each letter subtending about 0.35° horizontally and 0.52° vertically. A string of seven ampersands (&&&&&&&) displayed in either red or green color at fixation, and subtending about 2.46° horizontally and 0.52° vertically was used as the target. All stimuli were displayed against a black background.

### Design and Procedure

Each trial began with a 1000 ms central fixation screen (a white asterisk), followed by a memory set consisting of five digits presented at fixation. In the high WM load block, the digit sets were random sequences of five different digits presented for 2000 ms. In the low WM load block, the digit sets consisted of the same digit repeated five times and presented for 1000 ms. The presentation durations of the low and high memory load sets allowed participants to rehearse the set at least once before presentation of the Stroop-priming trials^1^. Participants had to memorize the set until the end of the trial. The order of high and low WM load blocks was counterbalanced between participants. The memory set offset was followed by a 500 ms blank screen, and then by two, three, or four consecutive Stroop-priming trials (see below for details). After the final Stroop-priming trial, a single memory probe digit was presented for 5000 ms or until response, and participants were asked to press the “1” or “2” keys with the middle and index fingers of their left hand to indicate whether or not the digit probe had been present in the memory set for that trial. Key allocations were counterbalanced between participants. On half of the WM trials, the digit probe was present in the set, with equal proportions of each digit position in the memory set. On the other half of the trials, the probe was absent in the memory set. A 500-ms feedback tone was presented following an incorrect response to the memory probe. After presentation of the memory set, and before the memory probe, the Stroop-priming task was presented.

In order to encourage active rehearsal of the memory set during the entire trial, the presentation of the memory probe was made unpredictable, by varying the number of Stroop-priming trials (see also De Fockert et al., 2001, for a similar procedure). Either two, three or four priming trials were presented during each WM trial. Each Stroop-priming trial began with a 500 ms central fixation (^∗^), followed by the prime word GREEN or RED presented in white uppercase letters for 100 ms at fixation. The prime display offset was followed by a 900 ms blank screen, followed by the target stimulus (thus resulting in a prime-target SOA of 1000 ms), which consisted of a series of seven ampersands displayed in either green or red color at fixation, which remained visible until a response had been made. The participants indicated the color (red or green) of the ampersands by pressing with the index and middle fingers of their right hand the “n” and the “m” keys on the computer keyboard. Both keys were labeled GREEN and RED (in green and red ink, respectively), with key-label allocations being counterbalanced between participants. A response to the color target triggered the next Stroop-priming trial, or the memory probe display. The prime–target pairings were incongruent (i.e., RED–green) on 80% of the trials and congruent (i.e., RED–red) on the remaining 20% of the trials. Before starting the experimental trials, participants were explicitly informed about the differential proportion of incongruent and congruent prime-target pairings trials, and were actively encouraged to capitalize on the predictive information provided by the prime word to optimize their performance.

Participants took part in a single session lasting about 25 min, consisting of 24 practice trials (12 for each WM load condition) followed by two experimental blocks, one with low and one with high WM load (order counterbalanced between participants). Participants were informed of the load type at the start of each block, and a break interval was included between experimental blocks. Participants completed 30 WM trials of each load condition, with each WM trial containing either two, three, or four Stroop-priming trials. There were 10 WM trials that had 2 Stroop-priming trials, 10 WM trials with 3 Stroop-priming trials, and 10 WM trials with 4 Stroop-priming trials, the order of which was randomized. From the 90 Stroop-priming trials of each WM load block, 72 were incongruent (80%), and 18 were congruent (20%). Within each of these two trial sets, the target was displayed either in red or in green on the same number of trials. The participants initiated each WM load block by pressing the space bar on the computer keyboard. Once a WM block was initiated, it ran to completion, so that the participants could rest only between blocks.

## Results

The analyses of responses to the WM probe confirmed that our load manipulation was effective in loading WM. Mean correct WM probe response times were reliably faster in the low WM load condition (*M* = 1404 ms) compared to the high WM load condition [*M* = 1909 ms; *t*(25) = 7.99, *p* < 0.001; *d* = 1.57]. Mean accuracy rates were also significantly higher in the low (*M* = 0.97) than in the high WM load condition [*M* = 0.94; *t*(25) = 2.46, *p* < 0.021; *d* = 0.48].

For the analysis of Stroop-priming responses, trials with target responses that were incorrect (2.58%) or faster than 200 ms (1.64%) were excluded. In addition, only trials on which the WM response was correct were included in this analysis^2^. Mean correct RT and error rate were computed for each participant as a function of congruency condition and WM load (see **Table 1**), and entered into two Analyses of Variance (ANOVAs), with prime-target congruency (congruent, incongruent) and WM Load (low, high) as within subjects factors. The ANOVA on error rates showed no significant effects.

**Table 1:** Mean reaction times (in milliseconds), and error percentages (in %) as a function of Working Memory Load (High vs. Low WM load), and Prime-target Congruency in the Stroop-priming task (Congruent vs. Incongruent).

	Prime-target Congruency	
	Congruent	Incongruent
Working Memory Load		
Low load	599 (131.4)	534 (102.3)
	2.2 (3.6)	2.1 (3.3)
High load	543 (118.1)	568 (115.2)
	1.5 (3.2)	1.6 (3.1)

*Standard deviation in brackets*.

The RT ANOVA revealed a main effect of congruency [*F*(1,25) = 6.33, *p* = 0.019, η^2^ = 0.20], such that responses to the target in the Stroop-priming task were faster on the incongruent than on the congruent trials (i.e., a reversed Stroop effect). The main effect of WM load was non-significant (*F* < 1). The key finding was a significant interaction between WM load and prime-target congruency [*F*(1,25) = 23.83, *p* < 0.001, η^2^ = 0.49]. As expected, a significant reversed Stroop effect was found when the attention task was performed under a low WM load, such that target responses were reliably faster (by 65 ms) on the incongruent than on the congruent trials [*t*(25) = 4.66, *p* < 0.001; *d* = 0.51]. In clear contrast, a standard Stroop interference effect was found when the Stroop-priming task was performed under high WM load, such that target responses were reliably slower (by 25 ms) for incongruent than for congruent prime-target pairings [*t*(25) = 2.46, *p* = 0.021; *d* = 0.21].

## Discussion

Our study was aimed to examine whether WM plays a role in the strategic control of selective attention. Using a version of the Stroop task, we found that participants’ ability to strategically utilize likelihood information about the nature of upcoming task events is significantly impaired when WM is loaded by an unrelated task. Participants successfully used the knowledge that incongruent prime-probe pairings were four times more likely than congruent pairings when the concurrent WM task had low load, and responded significantly faster on incongruent than congruent probes. This finding replicates previous demonstrations of expectancy-based strategic effects in the Stroop task (Froufe et al., 2009; see also Merikle and Joordens, 1997; Daza et al., 2002). Our novel finding was there was no such strategic benefit when WM was highly loaded, and instead a standard Stroop effect was found with faster responses to congruent than incongruent probes.

Recent semantic priming studies (Heyman et al., 2014; Hutchison et al., 2014) had shown that WM load can disrupt the use of controlled strategies like expectancy generation. In these studies, however, both the standard semantic priming effect and expectancy effects would lead to better performance for semantically related prime and probe information, making it difficult to distinguish between those possible underlying mechanisms. In the present study, we used a version of the Stroop task that allowed us to obtain qualitatively different (e.g., opposite) priming effects resulting from strategic (controlled) vs. non-strategic (automatic) processing of the relevant information (the prime word). As there are only two possible colors and the incongruent prime-target pairings are much more frequent than the congruent ones, the intelligent strategy is to expect that the target color on each trial will be the opposite to that of the prime word. Such a strategy would facilitate performance on the incongruent trials and slow performance on the congruent trials (i.e., a reversed Stroop interference). But under task conditions that make difficult (or impossible) the use of such a predictive strategy (e.g., subliminal presentation of the prime word) a standard Stroop interference effect is found (see for example Merikle and Joordens, 1997; Daza et al., 2002). Our findings show that loading WM during the Stroop task has a similar effect.

The reversed Stroop-priming effect under low WM load in our study replicates that usually observed with this task in healthy young adults under conditions that maximize a controlled processing of the prime word, such as presenting the prime for a relatively long duration and unmasked, and/or using a relatively long prime-target SOA (e.g., Merikle and Joordens, 1997; Daza et al., 2002; Froufe et al., 2009). In clear contrast, when the Stroop task was performed under high WM load, the opposite result was found, with target responses being reliably slower on incongruent than on congruent trials. This latter result replicates that previously observed by Froufe et al. (2009) in aged people with Alzheimer dementia, as well as the (supposedly automatic) Stroop interference that is usually found in healthy young adults under task conditions that minimize a controlled (strategic) processing of the prime stimulus, such as presenting the prime below an awareness threshold, and/or using a short prime-target SOA (e.g., Merikle and Joordens, 1997; Daza et al., 2002).

Note that under high WM load, participants were significantly slower to respond to incongruent trials versus congruent probes, even though the former were greatly more probable to occur than the latter. As the probability with which a condition occurs in the context of an experiment has a strong effect on response times (Hyman, 1953), the finding that this fundamental effect was reversed when participants were in the high WM load condition further suggests that we are dealing with a particularly robust effect.

The current results fit fairly well with Braver et al.’s (2007) distinction between proactive and reactive mechanisms of cognitive control, which was originally proposed by Braver et al. (2007) to account for impaired cognitive control exhibited in schizophrenia patients and older adults. Proactive control involves maintaining goal information in an accessible state so as to direct attention toward goal-relevant stimuli and away from potential internal and external distractions. This form of cognitive control is effortful and preparatory in nature, as uses predictive cues to prepare for a response to a specific upcoming target. Reactive control is a backward-acting process that is automatically triggered by target onset and involves retrieving prior contextual (e.g., goal) information from long-term memory. In contrast to proactive control, the reactive form of control does not require continuous effort or monitoring of the environment, but instead involves using a target stimulus to retrieve appropriate actions from long-term memory. The lack of a reversed Stroop-priming effect as a result of engaging in a high WM load in the present study could reflect an inability of participants to effectively represent and update the task instructions in WM until the target appeared.

Many previous studies examining the role of WM in selective attention have used verbal memory tasks similar to that in the present research (i.e., retaining digit-sets) to manipulate WM load. Such a WM task could encourage participants to use verbal coding processes (e.g., sub-vocal rehearsal) to maintain the stimulus set in an active state during the attention (Stroop-priming) task. It is not implausible that such verbal coding processes were more necessary for maintaining random series of different digits (high load) than several repetitions of a same digit (low load). Based on this line of argument, one could argue that relative to a low load, a high load in our verbal WM task could produce a greater interference with the verbal generation of the opposite name (e.g., “red”) given the written prime word (e.g., GREEN), thus explaining the elimination of the reversed Stroop effect observed under high WM load.

Whereas we cannot completely rule out the involvement of purely verbal interference effects from the verbal WM task in our study, several observations seem to be pertinent here. First, there is ample evidence that manipulations of control cognitive load other than verbal WM reliably affect performance on selective attention tasks (e.g., De Fockert et al., 2010; Chao, 2011). The effect of WM load on selective attention also persists even when the phonological loop is loaded by overt rehearsal in both high and low WM conditions (e.g., Lavie et al., 2004), or when the WM and selective attention tasks show little overlap in terms of stimulus content (e.g., Lavie et al., 2004; De Fockert and Bremner, 2011; Heyman et al., 2014). For example, and of more relevance for the present study, by using a non-verbal WM task (remember an easy vs. complex dot pattern), Heyman et al. (2014) have recently demonstrated that expectancy-based strategic processes underlying a semantic priming task were ineffectual under a high load in the non-verbal WM task. It then appears that the effect of WM load on selective attention is largely domain-general (attention control resources) rather than domain-specific. This conclusion would also be consistent with the executive attention model of WM proposed by Engle and Kane (2004) and Kane et al. (2007), which states that imposing a high WM load has the same effect on selective attention as having a low WM capacity, as attention control mechanisms are more efficient in individuals with greater WM capacity, compared to those with more limited WM resources. In either case, an interesting matter for future research would be to explore whether performing a non-verbal WM task under high load could also prevent or disrupt the implementation of expectancy-based strategies in our Stroop-priming task.

Secondly, to the extent that maintaining a high load verbal memory set like 57394 could induce a greater interference with the verbal generation of the opposite name in the Stroop task, than maintaining a low load verbal memory set like 22222, participants’ performance in the Stroop-priming task in terms of speed and/or accuracy should be worse under high than under low verbal WM load. Yet, we found no main effect of the WM load factor in our study, as both the speed and accuracy of responses to the Stroop task were highly similar under high and low WM load conditions.

Finally, verbal interference by the high WM load in our study led to a reversal of the Stroop-priming effect, from a reversed Stroop to a standard Stroop effect. Presumably, if verbal rehearsal of the WM set prevented processing of the prime word, then the Stroop-priming effect should be eliminated rather than reversed. The finding of a standard interference Stroop effect under high WM load thus suggests that the prime words were processed, but that strategic use to predict the upcoming response was prevented.

One could also argue that the elimination of the strategic priming effect under a high WM load is due to a general (non-specific) effect of increased cognitive control in that load condition, rather than to a more specific effect of WM load on selective attention. Note, for example, that the effect of increasing distractibility under conditions of high load are not limited to WM, as dual-task performance have similar effects on the magnitude of distractor effects in selective attention (e.g., Lavie et al., 2004, Experiments 4 and 5). Indeed, previous work using a Stroop-priming task like the one we used (Merikle and Joordens, 1997; Experiment 1B) has reported a similar pattern of opposite priming effects (reversed vs. standard Stroop effect) across conditions of focused vs. divided attention, respectively. Importantly, Merikle and Joordens (1997) found a reliable main effect of the attention manipulation, such that the divided-attention participants responded significantly slower on the Stroop task than the focused-attention participants. This result pattern suggests that participants’ performance in the Stroop-priming task significantly suffered as a result of engaging in a dual task (divided attention), and leaves open the possibility that the pattern of Stroop effects was in part driven by differences in general task difficulty. By contrast, as noted above, no main effect of the WM load factor was found in our study, with target responses in the Stroop task being highly similar in terms of both speed and accuracy under high and low working load conditions. Consequently, the elimination of the strategic priming effect that we found under high WM load cannot be explained simply in terms of increased task difficulty^3^. In line with results reported by some previous studies using different measures of selective attention (e.g., De Fockert and Bremner, 2011), imposing a high WM load in our Stroop-priming task seems to have had a very specific effect on the effective implementation of expectancy-based strategic process on the relevant information.

## Conclusion

The present results replicate and extent previous work in demonstrating that a reduction in the availability of WM by engaging WM in an additional task of high load, can lead to less efficient strategic processing of task-relevant information (e.g., Froufe et al., 2009; Heyman et al., 2014; Hutchison et al., 2014). Under conditions of low WM load, participants were able to strategically process the prime word in order to anticipate the target color, thus leading to reliable reversed Stroop-priming. In contrast, high WM load induced non-controlled (automatic) processing of the prime word, thus resulting in an opposite, standard Stroop interference effect. Loading WM during selective attention not only interferes with the ability to resist concurrent interference from distracting information (e.g., Lavie et al., 2004), but also reduces the efficiency with which task-relevant information can be strategically used over time in selective attention.

## Ethics Statement

All participants gave written consent after the nature and the consequences of the experiment had been explained. The present study has been approved by the local Ethical Committee and was conducted in accordance with the Declaration of Helsinki.

## Author Contributions

All the authors contributed equally to: (i) The conception and design of the work as well as analysis and interpretation of data; (ii) Drafting the work or revising it critically for important intellectual content; (iii) Final approval of the version to be published; and (iv) All of them are agreed to be accountable for all aspects of the work in ensuring that questions related to the accuracy or integrity of any part of the work are appropriately investigated and resolved.

## Conflict of Interest Statement

The authors declare that the research was conducted in the absence of any commercial or financial relationships that could be construed as a potential conflict of interest.

## Footnotes

1 Previous work using the combined working memory/selective attention paradigm, has demonstrated that manipulations of working memory load do influence performance on both the memory the attention tasks in a similar way, regardless of whether the memory-sets under high and low load conditions are presented for either the same (e.g., De Fockert et al., 2001; De Fockert and Bremner, 2011) or different (e.g., De Fockert and Leiser, 2014) time exposures. Yet, adjusting the retention interval as a function of the level of load on working memory has indeed become a standard practice in the type of sandwich-task where a working memory task of varying loads is wrapped around an unrelated second task (see for example the recent study by De Fockert and Leiser, 2014, in which the digit-sets for the low and high WM conditions were presented for 1000 and 2500 ms, respectively). In our experience, not doing so can disrupt task performance on both tasks to an extent as to make the data hard to interpret for many participants. An additional reason for adjusting the retention interval as a function of the level of load on working memory was to reduce/minimize the differences between low and high load conditions in terms of general task difficulty, thus making the high load condition less demanding and the low load condition more demanding than they would otherwise be.

2 An additional ANOVA on correct target responses, which also included trials on which the response to the probe memory was incorrect, revealed an identical result pattern. Namely, whereas the main effect of WM load was non-significant (*F* < 1), there was a significant interaction between WM load and prime-target congruency [*F*(1,25) = 14.37, *p* < 0.001, η^2^ = 0.37]. Further analyses revealed a significant reversed Stroop effect when the attention task was performed under a low WM load [41 ms; *t*(25) = 2.93, *p* = 0.007; *d* = 0.28], and a standard Stroop interference effect when the attention task was performed under high WM load [31 ms; *t*(25) = 2.32, *p* = 0.029; *d* = 0.30].

3 Some previous work has shown that cognitive control/selective attention tasks can be impacted by temporal aspects of the task (e.g., Grosjean et al., 2001; Schmidt, 2016). One could thus argue that using different retention intervals used for the high and low load conditions could differentially affect the pace of the experiment. But several observations seem pertinent here. First, some previous studies using the combined working memory/selective attention paradigm either require participants to perform the WM task on every selective attention (priming) trial, or present a fixed number of priming trials on each WM trial for both high and low load conditions. But neither of these were the case in the present study. Rather, a variable number of either two, three, or four Stroop-priming trials were randomly presented during each WM trial, in order to make unpredictable the presentation of the memory probe. This procedure should minimize the probability that the high and low load conditions could induce different rhythmic responding biases. Second and even most important, the fact that the WM load manipulation had no reliable impact on the overall target responses in the Stroop-priming task makes it unlikely that the effect of load on the strategic Stroop effect can be attributed to the difference in task pace. Especially in the context of the strategic Stroop effect at test here, one may predict that the slower pace of the experiment in the high working memory load condition would make it more, rather than less likely for participants to strategically use the cue to predict the upcoming target, which is in contrast to what we found.

## References

[B1] Ahmed L. De Fockert J. W. (2012). Focusing on attention: the effects of working memory capacity and load on selective attention. *PLoS ONE* 7:1–11. 10.1371/journal.pone.0043101 PMC3429456 22952636

[B2] Braver T. S. Gray J. R. Burgess G. C. (2007). “Explaining the many varieties of working memory variation: dual mechanisms of cognitive control,” in *Variation in Working Memory*, eds Conway A. Jarrold C. Kane M. J. Miyake A. Towse J. N. (New York, NY: Oxford University Press), 76–106.

[B3] Chao H. F. (2011). Active inhibition of a distractor word: the distractor precue benefit in the stroop color-naming task. *J. Exp. Psychol. Human* 37 799–812. 10.1037/a0022191 21480743

[B4] Conway A. R. Tuholski S. W. Shisler R. J. Engle R. W. (1999). The effect of memory load on negative priming: an individual differences investigation. *Mem. Cognit.* 27 1042–1050. 10.3758/BF03201233 10586579

[B5] Daza M. T. Ortells J. J. Fox E. (2002). Perception without awareness: further evidence from a Stroop priming task. *Percept. Psychophys.* 64 1316–1324. 10.3758/BF03194774 12519028

[B6] De Fockert J. Ramchurn A. Van Velzen J. Bergström Z. Bunce D. (2009). Behavioural and ERP evidence of increased interference in old age. *Brain Res.* 1282 67–73. 10.1016/j.brainres.2009.05.060 19497314

[B7] De Fockert J. W. (2005). Keeping priorities: the role of working memory and selective attention in cognitive aging. *Sci. Aging Knowledge Environ.* 2005 pe34. 10.1126/sageke.2005.44.pe34 16267341

[B8] De Fockert J. W. (2013). Beyond perceptual load and dilution: a review of the role of working memory in selective attention. *Front. Psychol.* 4:287 10.3389/fpsyg.2013.00287 PMC3659333 23734139

[B9] De Fockert J. W. Bremner A. J. (2011). Release of inattentional blindness by high working memory load: elucidating the relationship between working memory and selective attention. *Cognition* 121 400–408. 10.1016/j.cognition.2011.08.016 21937032

[B10] De Fockert J. W. Leiser J. (2014). Better target detection in the presence of collinear ?ankers under high working memory load. *Front. Hum. Neurosci.* 8:281 10.3389/fnhum.2014.00821 PMC4196630 25352803

[B11] De Fockert J. W. Mizon G. A. D’Ubaldo M. (2010). No negative priming without cognitive control. *J. Exp. Psychol. Human* 36 1333–1341. 10.1037/a0020404 20854003

[B12] De Fockert J. W. Rees G. Frith C. D. Lavie N. (2001). The role of working memory in visual selective attention. *Science* 291 1803–1806. 10.1126/science.1056496 11230699

[B13] Engle R. W. Kane M. J. (2004). “Executive attention, working memory capacity, and a two-factor theory of cognitive control,” in *The Psychology of Learning and Motivation*, ed. Ross B. (New York, NY: Elsevier), 145–199.

[B14] Froufe M. Cruz I. Sierra B. (2009). (dis)Función ejecutiva en personas mayores con y sin Alzheimer: actuación estratégica basada en expectativas. *Psicológica* 30 119–135.

[B15] Gazzaley A. (2012). “Top-down modulation deficit in the aging brain: an emerging theory of cognitive aging,” in *Principles of Frontal Lobe Function*, 2nd Edn, eds Stuss D. T. Knight R. T. (New York, NY: Oxford University Press), 593–608.

[B16] Grosjean M. Rosenbaum D. A. Elsinger C. (2001). Timing and reaction time. *J. Exp. Psychol. Gen.* 130 256–272. 10.1037/0096-3445.130.2.256 11409103

[B17] Heyman T. Van Rensbergen B. Storms G. Hutchison K. A. De Deyne S. (2014). The influence of working memory load on semantic priming. *J. Exp. Psychol. Learn.* 41 911–920. 10.1037/xlm0000050 25329088

[B18] Hutchison K. A. Heap S. J. Neely J. H. Thomas M. A. (2014). Attentional control and asymmetric associative priming. *J. Exp. Psychol. Learn.* 40 844–856. 10.1037/a0035781 24548327

[B19] Hyman R. (1953). Stimulus information as a determinant of reaction time. *J. Exp. Psychol.* 45 188–196. 10.1037/h0056940 13052851

[B20] Kane M. J. Conway A. R. A. Hambrick D. Z. Engle R. W. (2007). “Variation in working memory capacity as variation in executive attention and control,” in *Variation in Working Memory*, eds Conway A. R. A. Jarrold C. Kane M. J. Miyake A. Towse J. N. (New York, NY: Oxford University Press), 21–48.

[B21] Kane M. J. Engle R. W. (2003). Working-memory capacity and the control of attention: the contributions of goal neglect, response com- petition, and task set to Stroop interference. *J. Exp. Psychol. Gen.* 132 47–70. 10.1037/0096-3445.132.1.47 12656297

[B22] Lavie N. De Fockert J. W. (2005). The role of working memory in attentional capture. *Psychon. Bull. Rev.* 12 669–674. 10.3758/BF03196756 16447380

[B23] Lavie N. Hirst A. de Fockert J. W. Viding E. (2004). Load theory of selective attention and cognitive control. *J. Exp. Psychol. Gen.* 133 339–354. 10.1037/0096-3445.133.3.339 15355143

[B24] Merikle P. M. Joordens S. (1997). Parallels between perception without attention and perception without awareness. *Conscious. Cogn.* 6 219–236. 10.1006/ccog.1997.0310 9245455

[B25] Ortells J. J. Noguera C. Álvarez D. Carmona E. Houghton G. (2016). Individual differences in working memory capacity modulates semantic negative priming from single prime words. *Front. Psychol.* 7:1286 10.3389/fpsyg.2016.01286 PMC5002416 27621716

[B26] Petersen S. E. Posner M. I. (2012). The attention system of the human brain: 20 years after. *Annu. Rev. Neurosci.* 35 73–89. 10.1146/annurev-neuro-062111-150525 22524787 PMC3413263

[B27] Schmidt J. R. (2016). Temporal learning and rhythmic responding: no reduction in the proportion easy effect with variable response-stimulus intervals. *Front. Psychol.* 7:634 10.3389/fpsyg.2016.00634 PMC4852197 27199861

[B28] Young A. W. Ellis A. W. Flude B. M. McWeeny K. H. Hay D. C. (1986). Face name interference. *J. Exp. Psychol. Human* 12 466–475. 10.1037/0096-1523.12.4.466 2946803

[B29] Zanto T. P. Gazzaley A. (2014). “Attention and ageing,” in *The Oxford Handbook of Attention*, eds Nobre A. C. Kastner S. (New York, NY: Oxford University Press), 927–971. 10.1093/oxfordhb/9780199675111.013.02

